# Effects of bone marrow-derived cells on monocrotaline- and hypoxia-induced pulmonary hypertension in mice

**DOI:** 10.1186/1465-9921-8-8

**Published:** 2007-01-30

**Authors:** William Raoul, Orianne Wagner-Ballon, Guitanouch Saber, Anne Hulin, Elisabeth Marcos, Stéphane Giraudier, William Vainchenker, Serge Adnot, Saadia Eddahibi, Bernard Maitre

**Affiliations:** 1 Unité INSERM TGU841 – Université Paris XII, Créteil, France; 2 Unité INSERM U362 – Institut Gustave Roussy, Villejuif, France; 3 Unité de Pneumologie – Hôpital Henri Mondor, AP-HP, Créteil, France; 4 Service de Physiologie – Hôpital Henri Mondor, AP-HP, Créteil, France; 5 Service de Toxicologie-Pharmacologie, Hôpital Henri Mondor, AP-HP, Créteil, France; 6 Service d'Hématologie – Hôpital Henri Mondor, AP-HP, Créteil, France

## Abstract

**Background:**

Bone marrow -derived cells (BMDCs) can either limit or contribute to the process of pulmonary vascular remodeling. Whether the difference in their effects depends on the mechanism of pulmonary hypertension (PH) remains unknown.

**Objectives:**

We investigated the effect of BMDCs on PH induced in mice by either monocrotaline or exposure to chronic hypoxia.

**Methods:**

Intravenous administration of the active monocrotaline metabolite (monocrotaline pyrrole, MCTp) to C57BL/6 mice induced PH within 15 days, due to remodeling of small distal vessels. Three days after the MCTp injection, the mice were injected with BMDCs harvested from femurs and tibias of donor mice treated with 5-fluorouracil (3.5 mg IP/animal) to deplete mature cells and to allow proliferation of progenitor cells.

**Results:**

BMDCs significantly attenuated PH as assessed by reductions in right ventricular systolic pressure (20 ± 1 mmHg vs. 27 ± 1 mmHg, *P *≤ 0.01), right ventricle weight/left ventricle+septum weight ratio (0.29 ± 0.02 vs. 0.36 ± 0.01, *P *≤ 0.03), and percentage of muscularized vessels (26.4% vs. 33.5%, *P *≤ 0.05), compared to control animals treated with irradiated BMDCs. Tracking cells from constitutive GFP-expressing male donor mice with anti-GFP antibodies or chromosome Y level measurement by quantitative real-time PCR showed BMDCs in the lung. In contrast, chronically hypoxic mice subjected to the same procedure failed to show improvement in PH.

**Conclusion:**

These results show that BMDCs limit pulmonary vascular remodeling induced by vascular injury but not by hypoxia.

## Background

Several reports suggests that bone marrow-derived cells (BMDCs) are able to colonize the adult lung following injury. In mice subjected to total body irradiation, transplantation of a single donor marrow-derived stem cell was followed by engraftment and differentiation to non-hematopoietic tissues, including lung tissue [1]. Following bleomycin-induced injury in mice, mesenchymal stem cells colonized the lung and differentiate into type 1 or type II pneumocytes [2,3]. In addition, human studies after hematopoietic stem cell transplantation or lung transplantation have shown significant chimerism, particularly in epithelial and endothelial cells [4-7]. However, these results have not always be confirmed by more recent articles using multiple methods of engraftment assessment [8].

Whether BMDCs participate in the growth, repair, or homeostasis of the lung vasculature and, consequently, in endothelial cell differentiation and vascular remodeling after vascular injury is still a matter of debate. Using the rat model of monocrotaline (MCT)-induced pulmonary hypertension, Zhao *et al*. [9] showed that bone marrow-derived endothelial like progenitor cells protected against pulmonary hypertension and pulmonary artery muscularization and this effect is enhanced when cells were transfected with eNOS gene. The presence of BMDCs in the pulmonary vasculature of mice exposed to chronic hypoxia, as well as in the smooth muscle coat of pulmonary arteries from patients with chronic obstructive pulmonary disease, has been considered evidence that progenitor cells contribute to pulmonary vascular remodeling [10,11]. Into the opposite, in a pneumonectomy model of lung growth in mice, Voswinckel et *al*. [12] found no evidence of endothelial cell engraftment and O'Neill and coll [13] failed to show any engraftment of BMDCs in a model of hypoxia-mediated angiogenesis in the mouse spinotrapezius muscle. Moreover, GM-CSF induces a 23% increased in angiogenesis without evidence of bone marrow cells contribution to endothelium remodeling.

In the present study, we investigated whether BMDCs injected to mice modified the development of PH induced either by exposure to chronic hypoxia or by treatment with the active MCT metabolite MCT pyrrole (MCTp). In order to determine whether PH alterations were due to protection of the pulmonary vasculature by BMDCs, we used quantitative real-time PCR to detect lung cell engraftment and measured lung eNOS expression in each experimental condition.

## Materials and methods

### Animals

Wild-type C57BL/6J mice (Charles River Laboratories) and transgenic eGFP C57BL/6-TgN mice (a gift from Genopole, Evry, France) were used at 6–8 weeks of age. Animal care and procedures were in accordance with institutional guidelines. Female mice were injected via the tail vein with BMDCs harvested from male eGFP transgenic mice treated by intraperitoneal injection of 3.5 mg 5-fluorouracil (TEVA Pharma, Courbevoie, France) 3 days before [14]. In a second set of experiments, female mice were injected via the tail vein with immunomagnetically purified Lin – BMDCs from eGFP male mice, cell sorted on their CD45 expression in order to purify CD45^-^lin^- ^or CD45^+^lin^- ^cells. The lineage markers used for depletion were B220, CD3, CD5, Gr1, Mac1 and Ter 119 as previously described.

### Pulmonary hypertension models

Mice were either exposed to chronic hypoxia (10 % FiO_2 _for 15 days as previously described) or given MCTp. MCTp treatment was previously demonstrated as able to induce pulmonary hypertension in rats, we then decided to test this approach in mice. Monocrotaline (MCT) (Sigma-Aldrich, Lyon, France) was converted to MCTp using the method of Mattocks *et al*. [15]. MCTp was dissolved in N, N-dimethylformamide (DMF)/RPMI 1640 just before use. Animals were anesthetized with isoflurane (Forene®, Abbott, Queenborough, UK) in a cylindrical chamber and given a single injection of MCTp (5 mg/kg) into a tail vein. In previous experiments, we found that this treatment was followed within 15 days by moderate pulmonary inflammation and remodeling of the small distal vessels.

### Study protocol

Mice were divided in seven groups: 1) vehicle-injected mice maintained in room air; 2) MCTp-treated mice; 3) MCTp-treated mice injected 3 days later with BMDCs; 4) MCTp-treated mice injected 3 days later by irradiated (50 Grays) BMDCs 5) hypoxia-exposed mice 6) hypoxia-exposed mice injected 3 days later with BMDCs; and 7) hypoxia-exposed mice injected 3 days later with irradiated BMDCs.

### Assessment of pulmonary hypertension

Right ventricular systolic pressure (RVSP) and the right ventricle/left ventricle+septum weight ratio (RV/LV+S) were measured as previously described [16]. After fixation and paraffin embedding, 5 μm-thick lung sections were cut and stained with hematoxylin-phloxine-saffron. In each mouse, 30 intraacinar vessels accompanying alveolar ducts or alveoli were examined by an observer blinded to the treatment.

### Assessment of donor cell colonization

For recipient of GFP marrow cells, immunohistochemistry was performed using rabbit polyclonal anti-GFP antibody (Abcam, Cambridge, UK), to detect donor cells. The final reaction product was visualized with 3,3'-diaminobenzidine (DAB, Sigma, St Louis, USA). Sections were counterstained with hematoxylin.

Quantification of murine Y chromosome in lung tissue was achieved by quantitative real-time polymerase chain reaction (PCR): first, DNA was extracted and purified from mouse lung, previously washed-out from blood, using the DNeasy tissue kit (Qiagen, Courtaboeuf, France), then real-time PCR was carried out on a 7700-sequence detection system with SYBR Green detection kit (Applied Biosystems, Foster City, USA), using the following PCR primers purchased from Invitrogen (Cergy Pontoise, France): Forward 5'-AGG TCC TGC TCC TTC CTT TTG- 3'; and Reverse 5'-GCT TTC TCC TTC CTG ACA CAC TAC A- 3'. Primer sequences were based on data published by Gubbay *et al*. [17] and established using Blast software on the *Mus musculus domesticus *sex-determining region (Sry) gene AF009519 (National Institutes of Health, NIH, Bethesda, USA). These primers amplify a 24-bp product. Standard curves were generated by serially diluting male mouse genomic DNA prepared from lung tissue.

### eNOS expression detection

Immediately after removal, the lungs were quickly frozen in liquid nitrogen. After thawing at 0°C, the tissues were sonicated in 0.1 mM PBS containing antiproteases (1 μM leupeptin and 1 μM pepstatin A) and CHAPS (20 mmol/L) and the homogenate was centrifuged at 3,000 *g *for 10 min at 5°C. Western Blot analysis was then performed as previously described [16]. Finally, eNOS immunoreactivity was quantified using a semi-automated image analysis device (GeneTools, Syngene). Results are reported in arbitrary units.

### Statistical analysis

All results are reported as means ± SEMs. One-way analysis of variance (Kruskal-Wallis) was performed, and the Mann Whitney test was used to identify significant between-group differences. *P *values ≤ 0.05 were considered statistically significant.

## Results

### Effects of bone marrow-derived cells on monocrotaline-induced pulmonary hypertension (PH)

**Figure 1:**
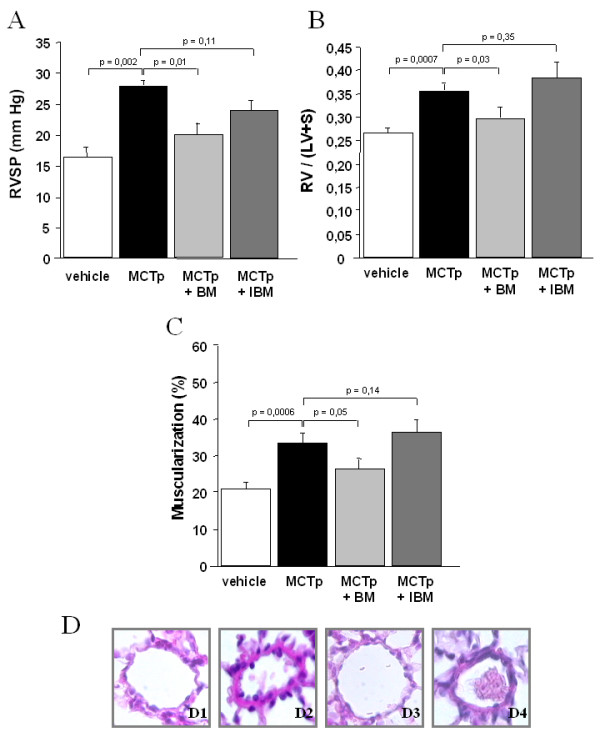
A. Ratio of right ventricle to left ventricle + septum weight [RV/(LV+S)] on day 15 in control untreated mice, MCTp-treated mice, MCTp-treated mice injected with bone marrow-derived cells (BMDCs), and MCTp-treated mice injected with irradiated BMDCs. Values are means ± SEM for 6 animals in each group except control mice (n = 15). B. Right ventricular systolic pressure (RVSP) on day 15 in control untreated mice, MCTp-treated mice, MCTp-treated mice injected with BMDCs, and MCTp-treated mice injected with irradiated BMDCs. Values are means ± SEM for 5 animals in each group except control mice (n = 8). C. Percentage of muscularized intraacinar vessels in lungs on day 15 in control untreated mice, MCTp-treated mice, MCTp-treated mice injected with BMDCs, and MCTp-treated mice injected with irradiated BMDCs. The percentage of pulmonary vessels in each muscularization category was determined by dividing the number of vessels in that category by the total number counted in the same experimental group. Values are means ± SEM for 5 animals in each group except control mice (n = 11). D. Representative panels of proximal artery remodeling. D1) normal artery in a control mouse; D2) MCTp-treated mouse with smooth muscle cell proliferation; D3) MCTp-treated mouse injected with bone marrow-derived cells (BMDCs) showing recovery of a nearly normal arterial morphology; D4) MCTp-treated mice injected with irradiated BMDCs showing no amelioration of remodeling or SMC proliferation. Sections were stained with hematoxylin-phloxine-saffron. Original magnification ×500.

**Figure 2:**
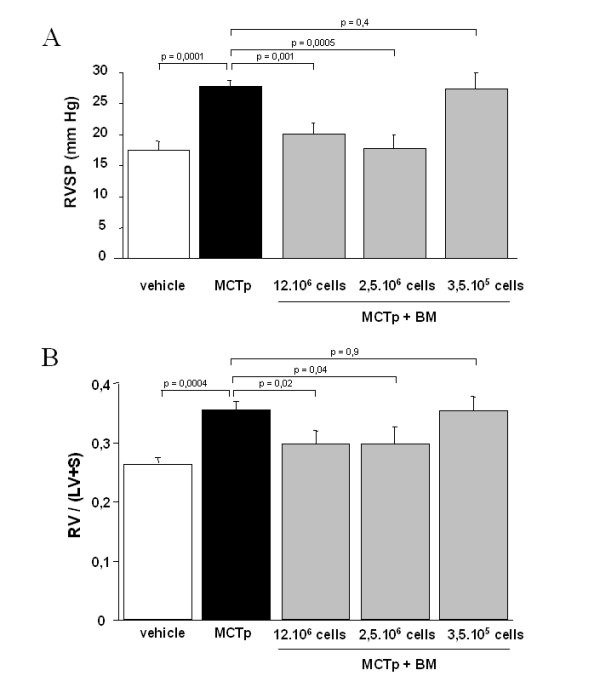
Effect of the number of bone marrow-derived cells on the increases in right ventricular systolic pressure (RVSP) and right ventricle to left ventricle + septum weight [RV/(LV+S)] after MCTp injection: 12·10^6 ^cells (2 male donor mice for 1 female recipient), 2.5·10^6 ^cells (1 male donor mouse for 1 female recipient), or 3.5·10^5 ^cells (1/2 male donor mouse sample for 1 female recipient). Values are means ± SEM for 5 animals in each group.

PH developed within 15 days of MCTp injection, as shown by a sustained increase in right ventricular systolic pressure (RVSP), RV/LV+S, and muscularization of distal pulmonary vessels, compared to vehicle-injected animals (Fig. 1). BMDCs injection 3 days after MCTp injection reduced the increases in RVSP and RV/LV+S in a dose-dependent manner. As shown in Fig. 2, a BMDCs number greater than 2.5·10^6 ^was needed to reduce RV pressure and hypertrophy, and no additional benefit was obtained by doubling the amount of BMDCs. Low BMDC doses (3.5·10^5 ^cells) had no effect on RVSP or RV/LV+S. Moreover control irradiated BMDCs had no effect on the development of PH in MCTp-treated mice (Fig. 1).

**Figure 3:**
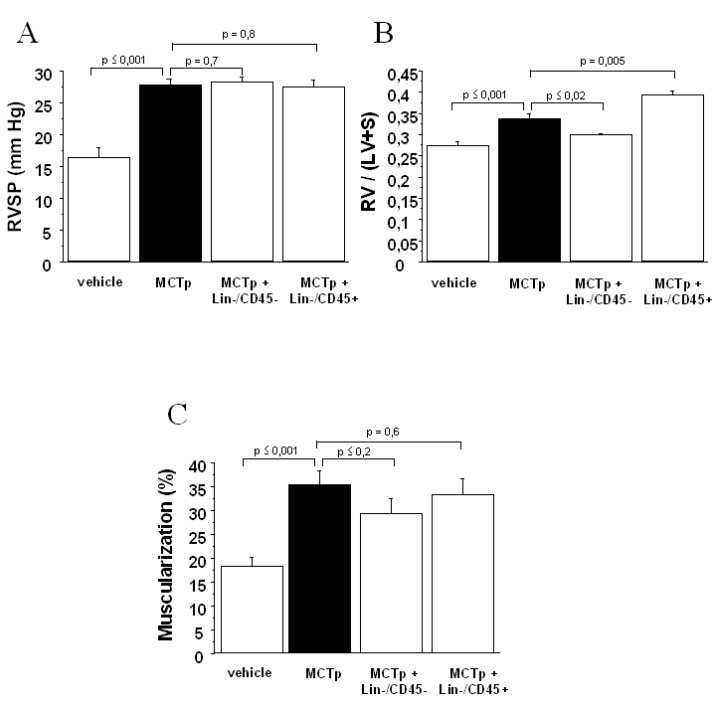
A. Ratio of right ventricle to left ventricle + septum weight [RV/(LV+S)] on day 15 in control untreated mice, MCTp-treated mice, MCTp-treated mice injected with Lin-/CD45- selected cells, and MCTp-treated mice injected with Lin-/CD45+ selected cells. Values are means ± SEM for 6 animals in each group. B. Right ventricular systolic pressure (RVSP) on day 15 in control untreated mice, MCTp-treated mice, MCTp-treated mice injected with Lin-/CD45- selected cells, and MCTp-treated mice injected with Lin-/CD45+ selected cells. Values are means ± SEM for 5 animals in each group. C. Percentage of muscularized intraacinar vessels in lungs on day 15 in control untreated mice, MCTp-treated mice, MCTp-treated mice injected with Lin-/CD45- selected cells, and MCTp-treated mice injected with Lin-/CD45+ selected cells. The percentage of pulmonary vessels in each muscularization category was determined by dividing the number of vessels in that category by the total number counted in the same experimental group. Values are means ± SEM for 6 animals in each group.

In order to determine whether non hematopoietic stem cells were responsible for this effect, we purified lineage-negative BMDC and injected either cell-sorted lin^-^CD45^+ ^and lin^-^CD45^- ^to MCTp-treated animals. Briefly, **1**0 000 flow-sorted and lin^-^CD45^- ^or 400 000 lin^-^CD45^+ ^male eGFP cells (which are equivalent as 2.5 × 10^6 ^cells for bone marrow reconstitution) were injected in MCTp-treated female mice. As shown on figure 3, lin^-^CD45^- ^cells injection reduced significantly the RV/LV + S ratio and the percentage of muscularized pulmonary distal vessels but RVSP were not statistically modified. This reduction of RV/LV + S ratio and of muscularization was not observed after lin^-^CD45+ treatment.

### Effects of bone marrow-derived cells on chronic hypoxia -induced PH

**Figure 4:**
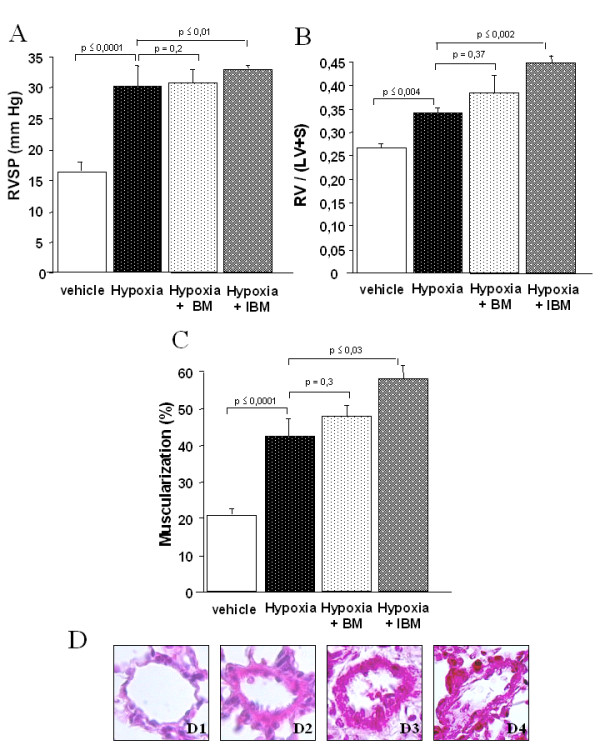
A. Ratio of right ventricle to left ventricle + septum weight [RV/(LV+S)] on day 15 in control untreated mice, hypoxia-exposed mice, hypoxia-exposed mice injected with bone marrow-derived cells (BMDCs), and hypoxia-exposed mice injected with irradiated BMDCs. Values are means ± SEM for 6 animals in each group except control mice (n = 11). B. Right ventricular systolic pressure (RVSP) on day 15 in control untreated mice, hypoxia-exposed mice, hypoxia-exposed mice injected with BMDCs, and hypoxia-exposed mice injected with irradiated BMDCs. Values are means ± SEM for 6 animals in each group. C. Percentage of muscularized intraacinar vessels in lungs on day 15 in control untreated mice, hypoxia-exposed mice, hypoxia-exposed mice injected with BMDCs, and hypoxia-exposed mice injected with irradiated BMDCs. The percentage of pulmonary vessels in each muscularization category was determined by dividing the number of vessels in that category by the total number counted in the same experimental group. Values are means ± SEM for 5 animals in each group except control mice (n = 10). D. Representative panels of proximal artery remodeling. D1) normal artery from a control mouse; D2) hypoxia-exposed mouse with SMC proliferation; D3) hypoxia-exposed mouse injected with BMDCs, with no evidence of recovery of normal arterial morphology; D4) hypoxia-exposed mouse injected with irradiated BMDCs, with no improvement in remodeling or SMC proliferation. Sections were stained with hematoxylin-phloxine-saffron. Original magnification ×500.

Mice exposed to 10% O_2 _for 15 days exhibited increases in RVSP and RV/LV+S similar to those observed in MCTp-treated mice. The percentage of muscularized pulmonary vessels was slightly higher in chronically hypoxic mice than in MCTp-treated mice (42.6 vs. 33.5 %, *P *< 0.05, Figs. 4 and 1, respectively). 2.5·10^6 ^BMDCs injection 3 days after exposure to hypoxia did not alter the development of PH as assessed by RVSP, RV/LV+S, and pulmonary vessel muscularization. Irradiated BMDCs increased the percentage of muscularized pulmonary vessels and the RV/LV+S index but do not significantly modified the RVSP. In view of these results, experiments with purified lin^-^CD45^+ ^and lin^-^CD45^- ^were not performed in this PH model.

### Detection of male bone marrow-derived cells in lungs from female recipient mice

**Figure 5:**
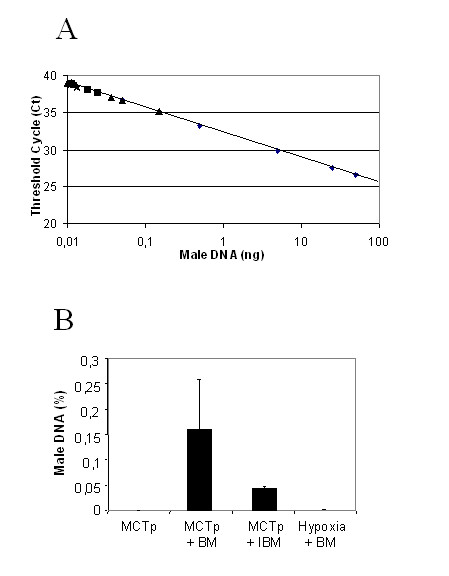
Panels A and B: Quantification of bone marrow-derived cells (BMDCs) engraftment in recipient mouse lung by real-time quantitative PCR. A) Relationship between the threshold cycle number and the percentage of male genomic DNA in the samples. The standard curve was generated by using samples containing 100 ng to 0.01 ng of male genomic DNA (losanges). Triangles: DNA from MCTp-treated mice injected with BMDCs 3 days later; squares: DNA from MCTp-treated mice injected with irradiated BMDCs 3 days later; stars : DNA from hypoxia-treated mice injected with BMDCs 3 days later. B) Histogram of the above-reported data showing the percentage of male genomic DNA in lung tissue from female mice treated with MCTp, MCTp followed by BMDCs (black bar), or MCTp followed by irradiated BMDCs (open bar) and hypoxia followed by BMDCs. Values are means ± SEM for 5 animals in each group.

To quantify bone marrow cell engraftment in female recipient mice, we performed quantitative real-time PCR on various total lung DNA samples. In DNA samples from female mice given both MCTp and male BMDCs, about 0.2% of total DNA was of male origin (Fig. 5, panels A and B), demonstrating that the graft was able to localize in the lung of these animals but in very few cells. In contrast, no or very low male DNA was detected in mice given both MCTp and irradiated male BMDCs.

### Detection of GFP-positive bone marrow-derived cells in lungs from recipient mice

**Figure 6:**
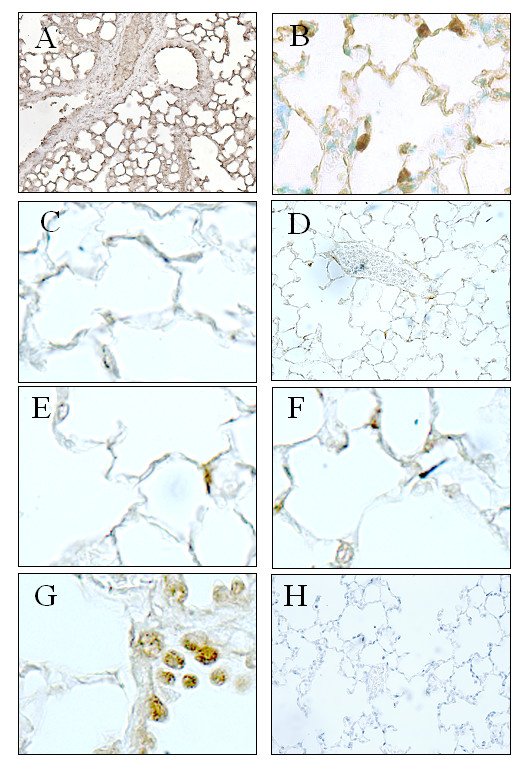
eGFP staining in representative lung panels. Representative panel of a control mouse lung (A and B) constitutively expressing GFP and wild-type mouse lung (C) injected with eGFP bone marrow-derived cells (BMDCs). Panels D, E, F and G are representative panels of recipient mouse lung 12 days after injection with eGFP BMDCs, which occurred 3 days after acute lung injury by a single administration of MCTp. Staining was detected in few cells in alveolar space, bronchial and distal arteries. Panel H. representative panel of recipient mouse lung exposed to hypoxia for 15 days and 12 days after injection with eGFP BMDCs: no staining was detected. Original magnification ×125 (A, D and H) ×500 (B, C, E, F and G).

Immunohistochemical analysis of lung sections from control eGFP-positive mice revealed strong immunostaining throughout the pulmonary parenchyma and vessels (Fig. 6 panel A and B). No staining was observed in lung sections from control C57BL/6 mice that did receive eGFP-positive BMDCs (Fig. 6, panel C). In contrast, lung sections from mice given both MCTp and eGFP-positive BMDCs (Fig. 6, panels D, E, F and G) showed staining in the bronchi, alveolar wall, and distal vessels 5 and 15 days after MCTp injection.

No eGFP immunostaining was detected in lung sections from chronically hypoxic mice given the same amount of eGFP-positive BMDCs (Fig. 6, panel H). Moreover, nor staining was noticed in MCTp-treated mice injected with eGFP-positive irradiated BMDCs (data not shown), nor was DNA detected in these mice using PCR approach. To investigate whether eGFP-positive cells were present in systemic organs, we performed immunohistochemical studies of liver and kidney tissue from MCTp- and hypoxia-exposed animals given eGFP-positive BMDCs. No staining was found in these organs (data not shown).

Using dual immunofluorescence with GFP antibody and CD31, CD45 or pan-cytokeratin antibodies, we were not able to indicate the phenotype of these engrafted cells which could be endothelial, hematopoietic or stromal cell types (data not shown).

### Effect of bone marrow-derived cells on eNOS expression

**Figure 7:**
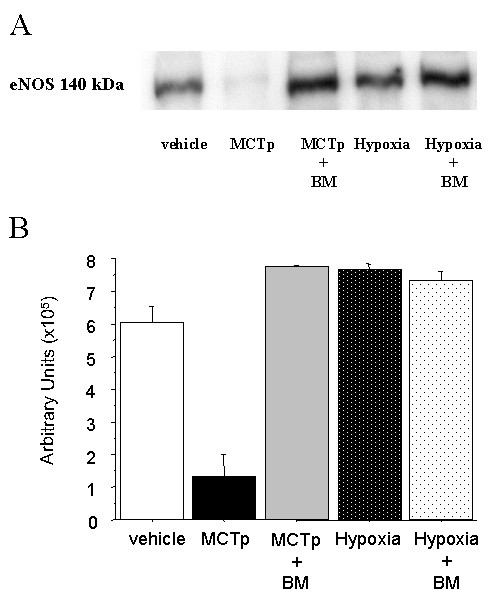
Western blot analysis of eNOS in lung tissues. A) Representative blot of eNOS protein. B) Quantification of eNOS immunoreactivity in lung homogenates from control untreated mice (vehicle), MCTp-treated mice, MCTp-treated mice injected with bone marrow-derived cells (BMDCs), hypoxia-exposed mice, and hypoxia-exposed mice injected with BMDCs. Values are means ± SEM for 5 animals in each group.

To evaluate the possible impact of BMDCs on the pulmonary endothelium lesions, we measured eNOS protein levels by immunoblotting in lungs from mice exposed to MCTp or hypoxia after BMDC injection. As shown in Fig. 7, eNOS protein levels were decreased in lungs from mice given MCTp, compared to controls, but not in mice given both MCTp and BMDCs. No alterations in lung eNOS protein levels were found in chronically hypoxic mice.

## Discussion

The present results show that administration of BMDCs protected against pulmonary hypertension in mice exposed to MCTp. The increase in lung eNOS expression suggest that BMDCs exerted protective effects on the endothelial cell layer of the pulmonary vessels but the detection of a low percentage of donor cells does not give conviction about the mechanisms of this beneficial effect. Our result show that BMDCs failed to improve hypoxia-induced PH, which, in contrast to MCTp-induced PH, is not preceded by acute endothelial injury.

Whether BMDCs contribute to the prevention or repair of pulmonary vascular injury is an important issue. In the present study, we used MCTp to induce pulmonary vascular injury and subsequent PH in mice. Because BMDCs injected after MCTp exposure protected against PH, it is likely that BMDCs exerted their beneficial effect by limiting the extent of the initial toxicity and vascular injury induced by MCTp.

We first investigated whether lung cell colonization could account for the protective effects of BMDCs. To minimize the risk of interpretation bias, we used different methods to detect donor cells in the lung. Using male DNA amplification by quantitative real-time PCR, we detected donor cells in mouse lungs 12 days after BMDC injection. However, the percentage of engrafted cells in lung is low as noted in the quantitative PCR results and the immunostaining results. In mice given BMDCs but no MCTp and in those given irradiated BMDCs and MCTp, no evidence of donor cell engraftment was obtained. This lack of detection has to be taken with caution and is probably dependent of the sensitivity of the techniques. Unfortunately we were not able to discriminate the phenotype of these engrafted cells with the single dual immunofluorescence assay used. It is very difficult to phenotype precisely cells in the lung and this explain for the most part the controversial results published about bone marrow cell plasticity in the lung. Others experiments such as confocal/deconvolution microscopy, FACS assays or even use of lineage-specific reporters should be performed to attend to phenotype cells in the alveolar region. Interestingly, using our MCTp model of PH, we found that lin^-^CD45^+ ^do not have a beneficial effect suggesting in our model a differentiation potential of non hematopoietic stem cells. Because of the literature focusing on the potential effect of endothelial progenitors, we did not perform Lin + cells experiment. Recently Cappocia and col [18] demonstrate that CD11b+ cells could be responsible of angiogenesis via a paracrine effect. In our model, we cannot rule out a potential beneficial effect of Lin + cells.

The importance of endothelial progenitor cells in protecting against PH has previously been addressed by two studies. Nagaya *et al*. [19] explored the effect of human cultured umbilical endothelial progenitor cells 3 days after MCT-induced injury in nude rats and showed a beneficial effect only when progenitor cells were transfected with adrenomedullin. More recently, Zhao *et al*. [9] reported a protective effect of rat endothelial progenitor cells in an MCT rat model, as well as an increase of this beneficial effect when cells were transfected with the e-NOS gene. Both studies confirmed an engraftment of progenitor cells but to a low level. In addition, others transfection studies using smooth muscle cells and fibroblasts gave also beneficial effects in these animal models [20,21]. All these results do not give evidence for a direct role of endothelial progenitor cells in the improvement of PH. In animals injected with MCTp, eNOS expression was markedly decreased. These results are consistent with previous studies showing that MCTp toxicity is associated with endothelial damage and reduced eNOS expression in the pulmonary vasculature. Interestingly, in animals treated with MCTp and injected with BMDCs, eNOS expression was not decreased and did not differ from that in control animals. These results again support the hypothesis that BMDCs limited MCTp-induced endothelial damage or improved endothelial repair.

To investigate whether BMDCs protected against the pulmonary vascular remodeling process, irrespective of the cause of PH, we also examined the effects of BMDC injection in mice with developing hypoxia-induced PH. Interestingly, BMDC injection did not improve PH in this model. Moreover, we were unable to detect lung cell engraftment or changes in eNOS expression in mice exposed to chronic hypoxia. Hypoxia-induced PH, in contrast to MCTp-induced PH, is not preceded by acute vascular injury. The dissimilar effects of BMDCs injection on PH in these two models are therefore consistent with the hypothesis that BMDCs exerted their protective effects and prevented PH by limiting the extent of MCTp-induced vascular injury or enhancing endothelial repair. Another reason for the lack of benefits in hypoxic mice may be related to the increase in circulating progenitor cells during hypoxia, as suggested by previous studies[22]. C-kit^+ ^cells were mobilized in calves subjected to hypoxia and were detected in peripheral blood and in remodeled pulmonary vessels [23]. In addition, Hayashida *et al*. [10] identified BMDCs in pulmonary arteries from mice subjected to BM transplantation followed by hypoxia exposure. These studies suggested that BMDCs might be mobilized during exposure to hypoxia and concomitant development of PH. Whether BMDCs contributed to or protected against hypoxia-induced PH could not be determined from these studies. In our study, BMDCs injection did not improve hypoxia-induced PH, suggesting a limited role as compared to MCTp-induced PH.

In conclusion, our study supports a beneficial effect of BMDCs infusion in a novel mouse model of pulmonary vascular injury and hypertension, model that was adapted from the well-known MCT rat model. The absence of benefits in hypoxia-induced PH suggests that initial vascular injury may be necessary to obtain a beneficial effect and to improve repair in the lung. However, the detection of a low engraftment of cells and the results obtained from BMDC purified stem cells injection in our model did not give insight into the mechanisms involved in this beneficial effect.

## Abbreviations

BMDCs : bone marrow derived cells

DAB : 3,3'-diaminobenzidine

eNOS : endothelial nitric oxide syntase

GFP : green fluorescent protein

GMCSF : granulocyte megacaryocyte colony stimulation factor

MCTp : monocrotaline pyrrole

PCR : polymerase chain reaction

PH : pulmonary hypertension

RVSP : right ventricular systolic pressure

RV/LV+S : right ventricle on left ventricle + septum

SMC : smooth muscle cell

## Competing interests

The author(s) declare that they have no competing interest

## Authors' contributions

WR carried out the experiments and drafted the manuscript, SG OWB and WV carried out the cell sorting analysis, GS, AH, EM carried out the toxicological preparation, hemodynamic and RT-PCR studies, SA and SE participated in the design of the study and helped to draft the manuscript, BM conceived of the study, and participated in its design and coordination and helped to draft the manuscript. All authors read and approved the final manuscript.

## References

[B1] Krause DS, Theise ND, Collector MI, Henegariu O, Hwang S, Gardner R, Neutzel S, Sharkis SJ (2001). Multi-organ, multi-lineage engraftment by a single bone marrow-derived stem cell. Cell.

[B2] Kotton DN, Ma BY, Cardoso WV, Sanderson EA, Summer RS, Williams MC, Fine A (2001). Bone marrow-derived cells as progenitors of lung alveolar epithelium. Development.

[B3] Ortiz LA, Gambelli F, McBride C, Gaupp D, Baddoo M, Kaminski N, Phinney DG (2003). Mesenchymal stem cell engraftment in lung is enhanced in response to bleomycin exposure and ameliorates its fibrotic effects. Proc Natl Acad Sci U S A.

[B4] Albera C, Polak JM, Janes S, Griffiths MJ, Alison MR, Wright NA, Navaratnarasah S, Poulsom R, Jeffery R, Fisher C, Burke M, Bishop AE (2005). Repopulation of human pulmonary epithelium by bone marrow cells: a potential means to promote repair. Tissue Eng.

[B5] Jiang S, Walker L, Afentoulis M, Anderson DA, Jauron-Mills L, Corless CL, Fleming WH (2004). Transplanted human bone marrow contributes to vascular endothelium. Proc Natl Acad Sci U S A.

[B6] Kleeberger W, Rothamel T, Glockner S, Flemming P, Lehmann U, Kreipe H (2002). High frequency of epithelial chimerism in liver transplants demonstrated by microdissection and STR-analysis. Hepatology.

[B7] Suratt BT, Cool CD, Serls AE, Chen L, Varella-Garcia M, Shpall EJ, Brown KK, Worthen GS (2003). Human pulmonary chimerism after hematopoietic stem cell transplantation. Am J Respir Crit Care Med.

[B8] Kotton DN, Fabian AJ, Mulligan RC (2005). Failure of bone marrow to reconstitute lung epithelium. Am J Respir Cell Mol Biol.

[B9] Zhao YD, Courtman DW, Deng Y, Kugathasan L, Zhang Q, Stewart DJ (2005). Rescue of monocrotaline-induced pulmonary arterial hypertension using bone marrow-derived endothelial-like progenitor cells: efficacy of combined cell and eNOS gene therapy in established disease. Circ Res.

[B10] Hayashida K, Fujita J, Miyake Y, Kawada H, Ando K, Ogawa S, Fukuda K (2005). Bone marrow-derived cells contribute to pulmonary vascular remodeling in hypoxia-induced pulmonary hypertension. Chest.

[B11] Peinado VI, Ramirez J, Roca J, Rodriguez-Roisin R, Barbera JA (2006). Identification of vascular progenitor cells in pulmonary arteries of patients with chronic obstructive pulmonary disease. Am J Respir Cell Mol Biol.

[B12] Voswinckel R, Ziegelhoeffer T, Heil M, Kostin S, Breier G, Mehling T, Haberberger R, Clauss M, Gaumann A, Schaper W, Seeger W (2003). Circulating vascular progenitor cells do not contribute to compensatory lung growth. Circ Res.

[B13] O'Neill TJ, Wamhoff BR, Owens GK, Skalak TC (2005). Mobilization of bone marrow-derived cells enhances the angiogenic response to hypoxia without transdifferentiation into endothelial cells. Circ Res.

[B14] Randall TD, Weissman IL (1997). Phenotypic and functional changes induced at the clonal level in hematopoietic stem cells after 5-fluorouracil treatment. Blood.

[B15] Mattocks AR, Jukes R, Brown J (1989). Simple procedure for preparing putative toxic metabolites of pyrrolizidine alkaloids. Toxicon.

[B16] Pascaud MA, Griscelli F, Raoul W, Marcos E, Opolon P, Raffestin B, Perricaudet M, Adnot S, Eddahibi S (2003). Lung overexpression of angiostatin aggravates pulmonary hypertension in chronically hypoxic mice. Am J Respir Cell Mol Biol.

[B17] Gubbay J, Collignon J, Koopman P, Capel B, Economou A, Munsterberg A, Vivian N, Goodfellow P, Lovell-Badge R (1990). A gene mapping to the sex-determining region of the mouse Y chromosome is a member of a novel family of embryonically expressed genes. Nature.

[B18] Capoccia BJ, Shepherd RM, Link DC (2006). G-CSF and AMD3100 mobilize monocytes into the blood that stimulate angiogenesis in vivo through a paracrine mechanism. Blood.

[B19] Nagaya N, Kangawa K, Kanda M, Uematsu M, Horio T, Fukuyama N, Hino J, Harada-Shiba M, Okumura H, Tabata Y, Mochizuki N, Chiba Y, Nishioka K, Miyatake K, Asahara T, Hara H, Mori H (2003). Hybrid cell-gene therapy for pulmonary hypertension based on phagocytosing action of endothelial progenitor cells. Circulation.

[B20] Zhao YD, Courtman DW, Ng DS, Robb MJ, Deng YP, Trogadis J, Han RN, Stewart DJ (2006). Microvascular regeneration in established pulmonary hypertension by angiogenic gene transfer. Am J Respir Cell Mol Biol.

[B21] Campbell AI, Zhao Y, Sandhu R, Stewart DJ (2001). Cell-based gene transfer of vascular endothelial growth factor attenuates monocrotaline-induced pulmonary hypertension. Circulation.

[B22] Ceradini DJ, Kulkarni AR, Callaghan MJ, Tepper OM, Bastidas N, Kleinman ME, Capla JM, Galiano RD, Levine JP, Gurtner GC (2004). Progenitor cell trafficking is regulated by hypoxic gradients through HIF-1 induction of SDF-1. Nat Med.

[B23] Davie NJ, Crossno JT, Frid MG, Hofmeister SE, Reeves JT, Hyde DM, Carpenter TC, Brunetti JA, McNiece IK, Stenmark KR (2004). Hypoxia-induced pulmonary artery adventitial remodeling and neovascularization: contribution of progenitor cells. Am J Physiol Lung Cell Mol Physiol.

